# The association between the frequency of soy protein intake and lower-limb muscle strength: a multicenter cross-sectional survey of adolescents in Mainland China

**DOI:** 10.3389/fpubh.2026.1924768

**Published:** 2026-09-14

**Authors:** Huijun Yan, Chao Kong, Guangtao Zhao, Min Zhang

**Affiliations:** 1 College of Physical Education and Health Management, Henan Finance University, Zhengzhou, China; 2 College of Physical Education, Liaoning Normal University, Dalian, China; 3 School of Physical Education, Chizhou University, Chizhou, China

**Keywords:** adolescents, cross-sectional study, frequency of soy protein intake, lower-limb muscle strength, standing long jump

## Abstract

**Background:**

Lower-limb muscle strength is a critical component of physical fitness in adolescents, yet the relationship between dietary protein sources and muscle function in this population remains underexplored. Soy protein, as a high-quality plant-based protein, may support musculoskeletal health, but evidence in Chinese adolescents remains limited.

**Methods:**

This multicenter cross-sectional survey included 24,602 adolescents (12,755 boys and 11,847 girls) from Mainland China. Frequency of soy protein intake was assessed by weekly consumption frequency and categorized into <2 times/week, 2–4 times/week, and >4 times/week. Lower-limb muscle strength was measured using standing long jump performance. Linear regression and logistic regression models were applied to examine the associations, with adjustments for age, BMI, screen time, breakfast frequency, and physical activity duration.

**Results:**

Participants with higher soy protein intake demonstrated better performance in the standing long jump across all groups. In the fully adjusted model, compared with the >4 times/week group, boys in the 2–4 times/week and <2 times/week groups showed reductions of −3.266 cm and −6.592 cm in jump distance, respectively; girls showed corresponding reductions of −4.717 cm and −5.992 cm; all *P* for trend <0.001. Logistic regression confirmed that lower soy protein intake was significantly associated with increased risk of failing the standing long jump standard, with the strongest association observed in girls consuming <2 times/week (OR = 1.79, 95% CI: 1.48–2.17).

**Conclusion:**

In this cross-sectional survey, increase the frequency of soy protein intake was positively associated with greater lower-limb muscle strength among Chinese adolescents. However, given the observational design, these findings do not establish causality. Further prospective studies are needed to confirm the temporal relationship and underlying mechanisms.

## 1 Introduction

Adolescence is a period of rapid muscle strength development and a critical window of opportunity (1). Lower-body muscle strength in adolescents is not only directly related to an individual’s athletic ability and level of physical activity. Still, it is also closely linked to bone health, metabolic function, and long-term risk of cardiovascular disease (2). A large body of research consistently shows that, whether assessed by grip strength, leg strength, or comprehensive measures of muscle strength, higher muscle strength is significantly associated with a lower risk of all-cause mortality (3, 4). The study found a nonlinear dose–response relationship between grip strength and the risk of death; for every 1-kilogram increase in grip strength, the hazard ratio (HR) was 0.97, indicating an approximately 3% reduction in the risk of death (5). However, over the past decade, the global decline in muscle strength among adolescents has become a major concern in public health. In Europe, a systematic review of 417,362 adolescents aged 6–16 across 20 countries showed that lower-body muscle strength (as measured by the standing long jump) trended upward until the 1980s and has declined steadily since then through 2023. The study further noted that scores across all categories of health-related physical fitness have shown a sustained decline among European adolescents (6). An analysis of single- and double-leg jump test data from adolescents between 2004 and 2022, conducted by the German DONALD cohort study, found that jumping parameters—as reflected in strength—declined significantly over time. The decline in athletic performance over the past nearly two decades is primarily attributed to a decrease in muscle strength (7). A French study based on five cross-sectional surveys of 53,314 children and adolescents aged 6–16, conducted between 1999 and 2023, also confirmed a significant downward trend in standing long jump performance, with a standardized effect size of −0.08 per decade, equivalent to a decrease of 2.1 cm (8). In China, data from the five national surveys on students’ physical fitness and health conducted between 2000 and 2019 show that although students’ body weight has continued to increase, their upper- and lower-body muscle strength has declined significantly since 2010 (9). Recent studies have also shown that muscle strength among Chinese adolescents has generally declined, with a particularly significant decline in lower-body strength. This issue warrants attention and consideration, and efforts should be made to identify the factors affecting muscle strength to improve muscle strength levels among adolescents (10, 11). This shows that the decline in lower-body muscle strength among adolescents is not an isolated phenomenon in any single country or region, but rather a global health challenge.

Muscle strength in adolescents is influenced by a combination of factors, including physical activity levels, sedentary behavior, sleep quality, and dietary nutrition (12, 13). Among the many influencing factors, the link between dietary protein intake and muscle health is receiving increasing attention. As the primary component of muscle protein synthesis, protein plays an irreplaceable role in the growth and development of skeletal muscle in adolescents (14). A study revealed a significant association between dietary protein intake and indicators of skeletal muscle health in adolescents, with marked gender differences observed (15). Among plant-based protein sources, soybeans and soy products have attracted significant attention due to their high-quality amino acid profile and abundance of bioactive compounds (16). Soy protein not only contains the amino acids required by the human body, but is also rich in soy isoflavones, which can regulate the expression of ubiquitin-specific proteases through the estrogen receptor *β* pathway, thereby promoting skeletal muscle growth under specific physiological conditions (17). A study involving 29,525 Chinese adults found that regular consumption of soy products was positively associated with grip strength. Compared with those who consumed soy products less than once a week, those who consumed them ≥4 times a week had a 36.2% lower risk of low grip strength (OR = 0.638). The study suggests that soy product consumption may have a protective effect on muscle health (18). Although there is a relatively large body of research on soy protein among adults and athletes, studies focusing on the general adolescent population—particularly those examining dietary intake rather than supplementation—remain very limited.

Against this backdrop, conducting a large-sample, multicenter study in mainland China on the association between soy product intake and lower-body muscle strength among adolescents is of significant practical importance. On the one hand, China is one of the world’s largest producers and consumers of soybeans, and soy products occupy an important place in traditional Chinese dietary culture; exploring the relationship between soy product intake and muscle health in adolescents can provide localized evidence for the development of dietary guidelines for adolescents tailored to China’s specific circumstances. On the other hand, previous studies have largely been limited to specific regions or single centers, resulting in samples that are not representative and cannot fully capture the true association between soy product consumption patterns and muscle strength among adolescents across different regions and socioeconomic backgrounds in China (19). A nationwide multicenter survey design can effectively overcome the limitations of single-center studies. By including adolescent samples from different regions and controlling for multiple confounding factors, this approach yields more robust and generalizable research conclusions. Furthermore, lower-body muscle strength (as measured by the standing long jump) better reflects adolescents’ daily activity capacity and overall physical function than upper-body grip strength (19). Using this indicator as an outcome variable provides a more comprehensive assessment of the impact of soy product intake on adolescents’ musculoskeletal health. Therefore, based on data from a nationwide, multicenter cross-sectional survey, this study systematically analyzes the cross-sectional association between dietary intake of soy products and lower-limb muscle strength among Chinese adolescents, providing a scientific basis for nutritional intervention strategies to promote musculoskeletal health in adolescents.

## 2 Methods

### 2.1 Participants

This study is a nationwide, multicenter, cross-sectional survey that used a multistage, stratified, cluster-randomized sampling method to select participants. It was conducted in mainland China from March to July 2024. Taking advantage of the schools’ administration of the National Student Physical Fitness Standards test, this study conducted independent tests and questionnaire surveys for the participants as part of this project. The sampling process was as follows: In the first stage, the country was divided into strata based on China’s geographic regions (East China, South China, North China, Central China, Southwest China, Northwest China, and Northeast China) and levels of economic development (high, medium, and low). Based on the 2023 Statistical Yearbook of Chinese Cities and the National Bureau of Statistics’ regional classification standards, using regional gross domestic product (GDP) as the indicator: >1 trillion yuan is classified as “high,” 300 billion to 1 trillion yuan as “medium,” and <300 billion yuan as “low” (20, 21). One to two prefecture-level cities were randomly selected from each stratum as survey sites, covering a total of 26 cities nationwide. The sampling frame for city selection comprised the complete list of all prefecture-level cities within each geographic–economic stratum. Within each stratum, cities were selected using computer-generated random numbers, thereby ensuring an unbiased and reproducible randomization procedure. See Table 1 for the specific distribution by city.

**Table 1:** Distribution of participant samples by city.

Geographic zone	City	Economic level	Number of schools	Number of participants
East China	Shanghai	High	3	1,245
	Nanjing	High	2	1,186
	Hefei	Moderate	2	889
	Suqian	Low	2	762
South China	Guangzhou	High	3	1,206
	Nanning	Moderate	2	854
	Shanwei	Low	2	818
North China	Tianjin	High	3	1,189
	Shijiazhuang	Moderate	2	809
	Taiyuan	Moderate	2	873
	Hohhot	Low	2	915
Central China	Wuhan	High	3	1,221
	Zhengzhou	Moderate	2	896
	Changsha	Moderate	2	818
	Ezhou	Low	2	904
Southwest China	Chengdu	High	3	1,232
	Chongqing	High	3	1,259
	Kunming	Moderate	2	770
	Liupanshui	Low	2	731
Northwest China	Xi’an	High	3	1,174
	Lanzhou	Moderate	2	751
	Urumqi	Moderate	2	766
	Guyuan	Low	2	694
Northeast China	Shenyang	High	3	1,143
	Changchun	Moderate	2	793
	Fuxin	Low	2	704
Total	26		60	24,602

In the second stage, within each survey site, junior high schools were randomly selected from urban and rural categories, with 2–3 schools chosen per site. Schools were selected at random from a complete list of all eligible schools registered with the local education authorities in each selected city. After the schools were assigned codes, they were randomly selected from the list using computer-generated random numbers, with stratified sampling by urban and rural areas. Ultimately, a total of 60 participating schools were selected nationwide. In the third stage, at each selected school, 2–3 complete classes were randomly selected from each of the first through third grades, and all students in those classes were included in the survey. All participants were 13–15-year-old junior high school students currently enrolled in school.

Sample size calculations were based on the requirements for analyzing associations between categorical variables in cross-sectional studies. Assuming a two-sided significance level of *α* = 0.05 and a power of (1 − *β*) = 0.80, and expecting a group-between difference in standing long jump performance of approximately 4.0 cm between the high-frequency soy product consumption group (≥5 times/week) and the low-frequency consumption group (<2 times/week), with a standard deviation of approximately 30.0 cm, each group would require a minimum of approximately 885 participants. Given that soy product consumption is categorized into three groups and that a multivariate regression analysis controlling for confounding factors is required, the design effect was set at 1.5. Taking into account a nonresponse or missing data rate of approximately 20%, the calculated minimum total sample size was approximately 15,000 participants. This study ultimately included 24,602 valid participants, exceeding the minimum sample size requirement and ensuring sufficient statistical power for stratified and subgroup analyses. All data were entered by two independent operators and cross-checked to ensure accuracy. Among the 24,602 participants ultimately included in the analysis, 12,755 were male (51.8%), and 11,847 were female (48.2%), with a mean age of 14.06 ± 0.82 years. The specific participant sampling process is shown in Figure 1.

**Figure 1:**
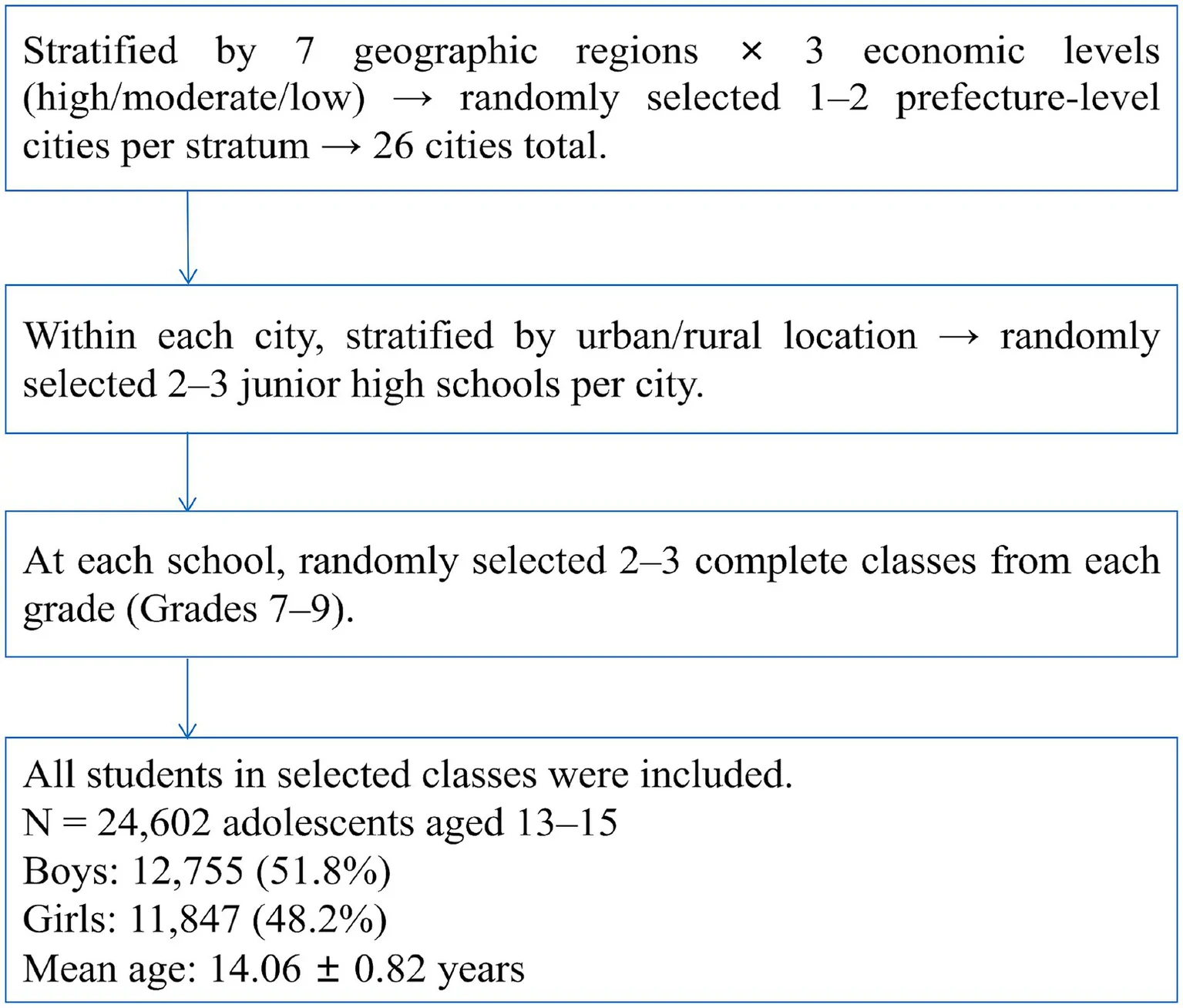
Sampling process for adolescent participants in Mainland China.

This investigation was carried out in line with the Declaration of Helsinki. Parental written informed consent was obtained, and each adolescent participant provided written assent before enrollment. Approved by the Human Ethics Committee of Chizhou University (CZXY-2026-TYXY-006).

### 2.2 Lower-limb muscle strength

In this study, the standing long jump was used as the assessment indicator for lower-limb muscle strength. The standing long jump assessment method strictly adhered to the National Student Physical Fitness Standards (Revised in 2014) (22). All participants completed the assessment during their school physical education classes. The testing area consisted of a standard level surface equipped with a standing long-jump mat (Model JH-1711). Participants stood with their feet naturally apart behind the takeoff line and pushed off the ground with both feet simultaneously to jump forward. No shuffling, running start, or consecutive jumps were permitted during the jump. The test result was recorded as the shortest vertical distance from the heel’s landing point to the takeoff line. Each student performed two consecutive jumps, and the best result was recorded, with results measured to the nearest 0.1 cm.

### 2.3 Frequency of soy protein intake

This study used a Food Frequency Questionnaire (FFQ) to assess the frequency of soy product consumption (23). The survey questions on the frequency of soy product consumption covered common types of soy products, including tofu, dried tofu, tofu skin, soy milk, soy-based milk, and fermented soy products. Participants reported their average consumption over the past month, with the following response options: “Rarely,” “Once a week,” “2–3 times a week,” “4–5 times a week,” “6–7 times a week,” and “≥7 times a week.” For statistical analysis, this study grouped soy product consumption frequency into three categories: <2 times per week (low-frequency group), 2–4 times per week (medium-frequency group), and ≥5 times per week (high-frequency group, serving as the reference group).

A study on the reliability and validity of a food frequency questionnaire for Chinese children and adolescents further demonstrates that this tool possesses good reliability and validity and can serve as a suitable instrument for surveying dietary and nutritional intake among adolescents. Before the formal survey, a pilot study was conducted with 120 students from one middle school to assess item comprehensibility and test test–retest reliability (test–retest correlation coefficient *r* = 0.78), indicating that the questionnaire has good reliability and validity.

### 2.4 Covariates

The covariates in this study included gender, age, screen time, duration of physical activity, breakfast, and body mass index.

Gender was collected via a questionnaire; age was calculated from the date of birth and the survey date and included in the analysis as a continuous variable.

In this study, screen time was assessed using a self-administered questionnaire to determine participants’ average daily total screen time over the past week, including time spent watching television and using electronic devices such as computers, tablets, and mobile phones. Screen time was categorized into three groups: <60 min/day, 60–120 min/day, and >120 min/day. This categorization is based on the internationally recognized 24-Hour Movement Behaviors Guidelines, which recommend that children and adolescents aged 5–17 limit recreational screen time to no more than 2 h (120 min) per day (24). Using 60 min and 120 min as cutoff points, the population can be divided into three categories: “below the lower limit recommended by the guidelines,” “in line with the guidelines,” and “above the upper limit recommended by the guidelines.” This facilitates the examination of the dose–response relationship between different levels of screen time and health outcomes. Furthermore, several previous large-scale cross-sectional studies of adolescents have used a similar three-group classification to analyze the association between screen time and physical fitness (25).

This study primarily examined participants’ average daily duration of moderate-to-vigorous physical activity (MVPA) over the past week, including physical education classes, recess, and after-school exercise. The average daily duration of physical activity was calculated based on the frequency of these activities and the duration of each session. In this study, the duration of physical activity was categorized into three groups: <30 min/day, 30–60 min/day, and >60 min/day. The World Health Organization (WHO) and guidelines from multiple countries explicitly recommend that adolescents accumulate at least 60 min of moderate-to-vigorous physical activity (MVPA) daily (26). Using 60 min as the threshold to distinguish between “meeting the standard” and “not meeting the standard,” the latest physical activity guidelines for children and adolescents in China, the United States, Canada, Australia, and the United Kingdom have all adopted this recommended standard (27).

Participants self-reported the number of days per week they ate breakfast over the past month. This study categorized breakfast frequency into three groups: ≤1 time per week, 2–3 times per week, and ≥4 times per week. This categorization is consistent with that used in numerous other studies (28).

In this study, Body Mass Index (BMI) was included as a continuous variable in the regression model. BMI was calculated based on height and weight. Height was measured using a standard height measuring device (accurate to 0.1 cm), and weight was measured using an electronic scale (accurate to 0.1 kg); BMI = weight (kg)/height (m)^2^.

### 2.5 Quality control

To ensure data quality, this study implemented quality control measures throughout the entire process. Prior to the survey, a standardized field survey operations manual was developed to ensure consistency in the testing process across different regions. A team of 35 graduate students serving as surveyors underwent centralized training and evaluation; only those who passed the evaluation were permitted to participate in the survey. Teams were deployed across seven regions nationwide, with each team consisting of 5 graduate students and 1 faculty member. Once the testing and evaluation process began, the seven teams simultaneously proceeded to their respective regions to carry out the testing and evaluation work. Each team was coordinated by one teacher, while overall coordination was handled by the project leader. The standing long jump test strictly adhered to the provisions of the National Student Physical Fitness Standards (Revised in 2014); each student was tested twice, and the best result was recorded. Testing equipment was calibrated at each survey site before use (22). The questionnaire used standardized instructions and was completed independently in the classroom. Interviewers verified the completeness of the questionnaires on-site and immediately clarified any questionable items with participants. These measures effectively controlled for information bias and measurement bias, ensuring the reliability of the research results.

### 2.6 Statistical analysis

All data analyses were performed using SPSS 26.0 software. All statistical tests were two-sided, and a *p*-value < 0.05 was considered statistically significant. Continuous variables are expressed as mean ± standard deviation (Mean ± SD), and categorical variables are expressed as percentages [*n*(%)]. The three groups were classified based on the frequency of soy product consumption (<2 times/week, 2–4 times/week, ≥5 times/week). One-way analysis of variance (ANOVA) was used to compare differences in continuous variables (age, height, weight, BMI, standing long jump) across groups. The chi-square test (*χ*^2^ test) was used to compare differences in the distribution of categorical variables (gender, screen time, duration of physical exercise, breakfast frequency) across groups. Assign values of 1, 2, and 3 to the three categories of soy product consumption frequency (<2 times/week, 2–4 times/week, >4 times/week), respectively, and include them as continuous variables in the regression model to calculate the *p*-value for trend.

Using standing long jump performance (a continuous variable) as the dependent variable, a multiple linear regression model was employed to estimate the association between soy product intake levels and standing long jump performance. The frequency of soy product consumption was included in the regression model as a categorical variable. Using ≥5 times per week (high-frequency group) as the reference group, two dummy variables were created: *X*₁ = 2–4 times per week (1 = yes, 0 = no) and *X*₂ = <2 times per week (1 = yes, 0 = no). In the trend test (*P* for trend), the three groups were assigned values of 1, 2, and 3, respectively, and included in the model as continuous variables. The analysis results are presented as estimates and their 95% confidence intervals (95% CI). Three models were constructed: the crude model (unadjusted), Model 1 (adjusted for age and BMI), and Model 2 (further adjusted for screen time, duration of physical activity, and breakfast frequency based on Model 1).

Passing Standards for the Standing Long Jump for Junior High School Students, Based on the Ministry of Education’s National Student Physical Fitness Standards (Revised in 2014) (22). The passing heights for boys are: 170 cm in 7th grade, 185 cm in 8th grade, and 170 cm in 9th grade; for girls, they are: 140 cm in 7th grade, 144 cm in 8th grade, and 146 cm in 9th grade. This study defined participants who fell below the standards for their respective grade and gender as “failing” and used this as the binary outcome variable for logistic regression analysis. Using standing long jump performance as the dependent variable, a binary logistic regression model was employed to analyze the association between insufficient soy product intake and the risk of failing to meet lower-limb muscle strength standards. Results are presented as odds ratios (OR) and their 95% confidence intervals (CI). The strategy for adjusting for covariates was consistent with that used in linear regression. All regression analyses were stratified by gender to control for potential interactions between gender and soy product intake.

## 3 Results

This study included a total of 24,602 adolescents aged 13–15 years, of whom 8,620 (35.0%) were in the high-frequency soy product intake group (>4 times per week), 12,966 (52.7%) were in the moderate-frequency group (2–4 times per week), and 3,016 (12.3%) were in the low-frequency group (<2 times per week). Differences in age, gender, screen time, duration of physical activity, breakfast frequency, height, weight, BMI, and standing long jump performance were all statistically significant among the three groups (all *p* < 0.001). Regarding the standing long jump, the high-frequency soy product group performed best (187.19 ± 30.09 cm), followed by the moderate-frequency group (184.94 ± 31.39 cm), with the low-frequency group performing the worst (184.67 ± 31.73 cm); the differences among groups were statistically significant (*F* = 15.600, *p* < 0.001). See Table 2 for specific results.

**Table 2:** Dietary soy protein intake among adolescents in Mainland China.

Categories	Frequency of soy protein intake			Total	*χ*^2^/*F*-value	*p*-value
	<2 times per week	2–4 times per week	>4 times per week			
*N*	3,016	12,966	8,620	24,602		
Age (years)	14.14 ± 0.81	14.05 ± 0.82	14.04 ± 0.82	14.06 ± 0.82	17.485	<0.001
Sex					215.163	<0.001
Boys	1,847 (61.2)	6,900 (53.2)	4,008 (46.5)	12,755 (51.8)		
Girls	1,169 (38.8)	6,066 (46.8)	4,612 (53.5)	11,847 (48.2)		
Screen time					390.010	<0.001
<60 min per day	1,374 (45.6)	6,416 (49.5)	5,210 (60.4)	13,000 (52.8)		
60–120 min per day	881 (29.2)	4,157 (32.1)	2,180 (25.3)	7,218 (29.3)		
>120 min per day	761 (25.2)	2,393 (18.5)	1,230 (14.3)	4,384 (17.8)		
Duration of physical activity					265.445	<0.001
<30 min per day	1,592 (52.8)	5,359 (41.3)	3,827 (44.4)	10,778 (43.8)		
30–60 min per day	1,165 (38.6)	5,709 (44)	3,243 (37.6)	10,117 (41.1)		
>60 min per day	259 (8.6)	1,898 (14.6)	1,550 (18.0)	3,707 (15.1)		
Breakfast					296.470	<0.001
≤1 times per week	246 (8.2)	499 (3.8)	315 (3.7)	1,060 (4.3)		
2–3 times per week	550 (18.2)	1,969 (15.2)	894 (10.4)	3,413 (13.9)		
≥4 times per week	2,220 (73.6)	10,498 (81)	7,411 (86)	20,129 (81.8)		
Height	166.33 ± 8.76	164.96 ± 8.59	164.42 ± 8.52	164.94 ± 8.61	55.093	<0.001
Weight	56.49 ± 11.79	54.06 ± 10.98	53.29 ± 10.59	54.09 ± 10.99	95.582	<0.001
Body mass index	20.36 ± 3.66	19.77 ± 3.21	19.62 ± 3.14	19.79 ± 3.25	57.536	<0.001
Standing long jump	184.67 ± 31.73	184.94 ± 31.39	187.19 ± 30.09	185.69 ± 31.00	15.600	<0.001

Univariate analysis (Table 3) showed that differences in standing long jump performance between different soy product intake frequency groups were statistically significant (all *p* < 0.001), both in the overall population and when stratified by gender. The trend indicated that a higher frequency of soy product intake was associated with better performance in the standing long jump. In the overall population, the high-frequency group (187.19 ± 30.09 cm) outperformed the medium-frequency group (184.94 ± 31.39 cm) and the low-frequency group (184.67 ± 31.73 cm).

**Table 3:** Univariate analysis of the association between frequency of soy protein intake and lower-limb muscle strength among adolescents in Mainland China.

Frequency of soy protein intake	*N*	Standing long jump		*F*-value	*p*-value
		Mean	Standard deviation		
Boys				46.370	<0.001
<2 times per week	1,847	197.32	30.82		
2–4 times per week	6,900	201.05	30.09		
>4 times per week	4,008	205.07	29.65		
Girls				98.157	<0.001
<2 times per week	1,169	164.69	21.08		
2–4 times per week	6,066	166.60	21.08		
>4 times per week	4,612	171.66	20.23		
Total				15.600	<0.001
<2 times per week	3,016	184.67	31.73		
2–4 times per week	12,966	184.94	31.39		
>4 times per week	8,620	187.19	30.09		

Multiple linear regression analysis (Table 4) showed that, both before and after adjusting for covariates, the frequency of soy product consumption was significantly and positively associated with standing long jump performance (*P* for trend < 0.001 in both cases). Using the high-frequency group (>4 times per week) as the reference, in Model 2 (adjusted for age, BMI, screen time, breakfast frequency, and duration of physical exercise), the average standing long jump distance in the moderate-frequency group was 4.040 cm lower (95% CI: −4.718 to −3.362) in the overall population, while the low-frequency group showed an average decrease of 6.439 cm (95% CI: −7.485 to −5.394). When stratified by gender, the average standing long jump distance in the moderate-frequency and low-frequency groups among male students decreased by 3.266 cm and 6.592 cm, respectively; among female students, it decreased by 4.717 cm and 5.992 cm, respectively. All of these differences were statistically significant (*p* < 0.001), indicating that insufficient intake of soy products is independently associated with reduced lower-body muscle strength. The dose–response trend test showed that for each one-level increase in the frequency of soy product consumption, standing long jump performance exhibited a significant upward trend (*P* for trend <0.001 in all models), indicating a dose–response relationship between soy product consumption and lower-body muscle strength in adolescents.

**Table 4:** Linear regression analysis of the relationship between frequency of soy protein intake and lower-limb muscle strength among adolescents in Mainland China.

Frequency of soy protein intake	Estimates (95% Confidence Interval)		
	Crude model	Model 1	Model 2
Boys			
>4 times per week	0.000 (Reference)	0.000 (Reference)	0.000 (Reference)
2–4 times per week	−4.017 (−5.187, −2.846)^a^	−3.693 (−4.778, −2.609)^a^	−3.266 (−4.349, −2.184)^a^
<2 times per week	−7.752 (−9.409, −6.095)^a^	−8.071 (−9.61, −6.531)^a^	−6.592 (−8.142, −5.042)^a^
*P* for trend	<0.001	<0.001	<0.001
Girls			
>4 times per week	0.000 (Reference)	0.000 (Reference)	0.000 (Reference)
2–4 times per week	−5.051 (−5.846, −4.257)^a^	−5.079 (−5.861, −4.297)^a^	−4.717 (−5.500, −3.934)^a^
<2 times per week	−6.969 (−8.302, −5.637)^a^	−7.059 (−8.372, −5.746)^a^	−5.992 (−7.317, −4.666)^a^
*P* for trend	<0.001	<0.001	<0.001
Total			
>4 times per week	0.000 (Reference)	0.000 (Reference)	0.000 (Reference)
2–4 times per week	−2.255 (−3.099, −1.412)^a^	−4.459 (−5.136, −3.781)^a^	−4.040 (−4.718, −3.362)^a^
<2 times per week	−2.522 (−3.807, −1.238)^a^	−7.780 (−8.817, −6.744)^a^	−6.439 (−7.485, −5.394)^a^
*P* for trend	<0.001	<0.001	<0.001

Model 1 controlled for age and BMI, while Model 2 controlled for screen time, frequency of breakfast, and duration of physical activity. “^a^” indicates *p* < 0.001.

Binary logistic regression analysis (Table 5) showed a significant association between insufficient intake of soy products and an increased risk of failing the standing long jump test (*P* for trend <0.001 in all cases). In Model 2 (adjusted for age, BMI, screen time, breakfast frequency, and duration of physical activity), compared with the high-frequency group (>4 times per week), the low-frequency group (<2 times per week) in the overall population had a 62% increased risk of failing to meet the standard (OR = 1.62, 95% CI: 1.45–1.83), and the risk increased by 21% in the moderate-frequency group (<2 times per week) (OR = 1.21, 95% CI: 1.11–1.32). Results from gender-stratified analyses indicated a dose–response relationship between soy product intake frequency and the risk of failing the lower-limb muscle strength test, with a stronger association among female than male students.

**Table 5:** Logistic regression analysis of the association between frequency of soy protein intake and lower-limb muscle strength among adolescents in Mainland China.

Frequency of soy protein intake	Odds ratio (95% confidence interval)		
	Crude model	Model 1	Model 2
Boys			
>4 times per week	1.00 (Reference)	1.00 (Reference)	1.00 (Reference)
2–4 times per week	1.12 (1.00–1.25)	1.10 (0.98–1.24)	1.07 (0.96–1.20)
<2 times per week	1.79 (1.55–2.07)^a^	1.66 (1.44–1.93)^a^	1.51 (1.30–1.75)^a^
*P* for trend	<0.001	<0.001	<0.001
Girls			
>4 times per week	1.00 (Reference)	1.00 (Reference)	1.00 (Reference)
2–4 times per week	1.47 (1.29–1.67)^a^	1.47 (1.29–1.67)^a^	1.41 (1.24–1.61)^a^
<2 times per week	2.00 (1.66–2.42)^a^	1.98 (1.64–2.4)^a^	1.79 (1.48–2.17)^a^
*P* for trend	<0.001	<0.001	<0.001
Total			
>4 times per week	1.00 (Reference)	1.00 (Reference)	1.00 (Reference)
2–4 times per week	1.10 (0.98–1.24)	1.25 (1.15–1.36)^a^	1.21 (1.11–1.32)^a^
<2 times per week	1.66 (1.44–1.93)^a^	1.80 (1.61–2.02)^a^	1.62 (1.45–1.83)^a^
*P* for trend	<0.001	<0.001	<0.001

Model 1 controlled for age and BMI, while Model 2 controlled for screen time, frequency of breakfast, and duration of physical activity. “^a^” indicates *p* < 0.001.

## 4 Discussion

This study found a significant positive association between the frequency of soy product consumption and lower-body muscle strength (measured by standing long jump performance) among adolescents aged 13–15 in mainland China. This association remained robust after adjusting for multiple confounding factors, including age, BMI, screen time, breakfast frequency, and duration of physical activity. These findings are generally consistent with previous research. A 5-year longitudinal study by Ng et al. (29) involving 436 adolescents in Malaysia confirmed a significant positive correlation between protein intake and muscle strength in male adolescents (*β* = 0.035, *p* = 0.016); however, the study did not analyze soy products as a specific source. This study is the report a dose–response relationship between the frequency of soy product consumption and lower-limb muscle strength in a nationally representative population. It may provide valuable insights and guidance for future muscle strength interventions among Chinese adolescents.

The positive association between soy product intake and lower-limb muscle strength observed in this study may involve multiple physiological mechanisms that act in concert at the molecular, cellular, and systemic levels. First, soybeans are a high-quality source of plant protein, rich in protein and containing all the essential amino acids required by the human body. Compared with other plant proteins, the amino acid composition of soy protein more closely matches human requirements, particularly because of its high leucine content among the branched-chain amino acids. Leucine is considered a key amino acid in the signaling pathways that stimulate muscle protein synthesis. Studies have confirmed that when soy protein and whey protein are matched for leucine content, there is no significant difference between the two in promoting muscle growth and strength development (30). A 2023 systematic review by Zare et al. included multiple randomized controlled trials and assessed the effects of soy protein supplementation on muscle adaptation, metabolism, and antioxidant status in active individuals and athletes. The results indicated that soy protein can increase lean body mass, enhance antioxidant status, and reduce oxidative stress (31). In terms of post-exercise recovery, soy protein supplementation can improve high-intensity running performance, increase maximum cardiac output, delay fatigue, and enhance isometric muscle strength (32). In addition, soy protein directly participates in the synthesis and repair of muscle protein by providing amino acid substrates. This evidence suggests that soy protein, as a high-quality dietary protein source, directly promotes net muscle protein synthesis by providing synthetic substrates and activating anabolic signaling pathways (33). Second, the isoflavones in soybeans may play a more complex and nuanced regulatory role in muscle metabolism. Soy isoflavones primarily include genistein, daidzein, and glycitein; because their chemical structures are similar to those of 17*β*-estradiol, they can bind to estrogen receptors *α* and *β*, thereby exerting estrogen-like activity (34). At the cellular level, studies have shown that soybean extract and its three major isoflavone aglycones exhibit anabolic activity in C2C12 myotubes by binding to estrogen receptors and regulating the expression of insulin-like growth factor-1 and myosin heavy chain (35). In animal experiments, Ogawa et al. (36) found that daidzin can downregulate the expression of ubiquitin-specific proteases via the estrogen receptor *β* pathway, thereby increasing sole muscle mass in young female mice. This finding reveals the molecular mechanism by which isoflavones maintain muscle mass by inhibiting protein degradation pathways. Furthermore, soy isoflavones have also been shown to play a significant role in counteracting inflammation-related muscle atrophy. Hirasaka et al. (37) found that isoflavones (including genistein and daidzein) can suppress the expression of the muscle-specific ubiquitin ligase MuRF1, which is induced by tumor necrosis factor-*α*, by activating the SIRT1 signaling pathway, thereby preventing the atrophy of C2C12 myotubes. In animal tumor models, dietary isoflavone supplementation significantly improves muscle wasting in tumor-bearing mice by inhibiting the expression of ubiquitin ligases mediated by the ERK signaling pathway (38). Further research has shown that daidzin can also mitigate skeletal muscle atrophy by regulating the AMPK/FOXO3a axis (39). Taken together, these findings suggest that soy isoflavones inhibit ubiquitin-proteasome-dependent protein degradation through multiple signaling pathways, may thereby protecting muscle mass during states of inflammation and catabolism.

It is worth noting that in this study, the strength of the association between soy product intake and muscle strength was higher among female participants (OR = 1.79) than among male participants (OR = 1.51); this may be related to the unique role of estrogen signaling pathways in female muscle metabolism. Estrogen exerts its regulatory effects in skeletal muscle through the estrogen receptors *α* and *β* (40). Since soy isoflavones preferentially bind to the estrogen receptor *β*, and the expression pattern of the estrogen receptor *β* in skeletal muscle may be gender-dependent, this may result in isoflavones exhibiting a stronger muscle-protective effect in women (41). In addition, estrogen deficiency impairs muscle protein synthesis, and the estrogen-like activity of isoflavones may partially compensate for this deficiency in women (42).

In addition, soy product intake may indirectly improve muscle strength by increasing satiety, reducing excessive energy intake, and decreasing fat accumulation. Soy products are rich in dietary fiber and protein, both of which enhance satiety and help maintain a healthy weight and body composition. In this study, the BMI of the group with high soy product intake was significantly lower than that of the group with low intake; this observation is consistent with the indirect mechanisms described above. Lower body fat levels are beneficial for muscle strength performance, as excessive fat tissue may increase the physiological burden of exercise and impair muscle efficiency.

This study has certain strengths. This study, which analyzed the relationship between adolescents’ intake of soy products and lower-limb muscle strength through a nationwide, multicenter survey with a large sample size, is highly representative. However, this study also has some limitations. First, as a cross-sectional study, it can only reveal an association between soy product intake and lower-limb muscle strength; it cannot establish a causal relationship. Further validation through longitudinal cohort studies or randomized controlled trials is needed. Second, the frequency of soy product intake was assessed using a self-reported food frequency questionnaire, which may be subject to recall bias. Additionally, the questionnaire collected only consumption frequency without quantifying the amount consumed per occasion, making it impossible to assess total intake. Third, although this study adjusted for multiple covariates, it cannot rule out residual confounding effects from other variables, including total energy intake, total protein intake, sleep duration, pubertal development, and detailed exercise intensity/type, and household socioeconomic status. Future prospective studies should incorporate comprehensive dietary assessments to more clearly elucidate the independent role of soy products in lower-limb muscle mass among adolescents. Fourth, this study included only 13–15-year-old middle school students; whether the findings can be generalized to adolescents in other age groups requires further verification. Furthermore, since the data in this study are cross-sectional, it is not possible to draw any definitive conclusions regarding causality. We must also consider the possibility that this relationship could hold in the opposite direction. For example, adolescents with greater lower-body muscle strength may be more likely to choose healthy foods, including consuming soy products more frequently. This is precisely why we have deliberately framed all mechanistic explanations as speculative.

## 5 Conclusion

Among adolescents aged 13–15 in mainland China, a higher frequency of soy product intake was significantly and positively correlated with better lower-body muscle strength (as measured by standing long jump performance). A dose–response relationship was observed between insufficient soy product intake and an increased risk of failing to meet the standing long jump standard, with the association being stronger among girls than among boys. The findings of this study provide a scientific reference based on large-scale data from mainland China for developing dietary intervention strategies to promote muscle health in adolescents.

## Data Availability

The raw data supporting the conclusions of this article will be made available by the authors, without undue reservation.
